# Immunopeptidome Landscape During *Brucella melitensis* Infection in Mice

**DOI:** 10.3390/ijms26188874

**Published:** 2025-09-12

**Authors:** Jing Jin, Yaming Sheng, Tingting Li, Kang Wang, Fanghao Geng, Yi Li, Jianfeng Gao

**Affiliations:** 1 College of Life Sciences, Shihezi University, Shihezi 832000, China; 18095982833@163.com (J.J.); 17690571196@163.com (T.L.); 20212006006@stu.shzu.edu.cn (F.G.); liyi12311206@163.com (Y.L.); 2 College of Life Sciences, The Chinese University of Hong Kong, Hong Kong 999077, China; 1155174033@link.cuhk.edu.hk; 3 Medical College, Shihezi University, Shihezi 832000, China; 15739338631@163.com

**Keywords:** *Brucella melitensis*, BMDCs, immunoprecipitation, mass spectrometry, immunopeptidome

## Abstract

Mouse bone marrow-derived dendritic cells (BMDCs) were infected in vitro with the recombinant *Brucella melitensis M5^+gfp^* strain. The immunopeptidome of *Brucella melitensis M5^+gfp^*, which presented peptides bound to MHC class II molecules on their surface, was isolated and characterized. BMDCs infected with *Brucella melitensis M5^+gfp^* were subjected to hypotonic lysis. The associated immunopeptidome was then isolated and characterized using co-immunoprecipitation (Co-IP) coupled with liquid chromatography–tandem mass spectrometry (LC-MS/MS). A total of 289 MHC-II-bound *Brucella melitensis M5^+gfp^* peptide sequences were identified, mapping to 183 distinct proteins. We successfully define the *Brucella melitensis M5^+gfp^* immunopeptidome presented by MHC-II on infected BMDCs. The source proteins of these peptides exhibited significant abundance and functional, structural, and pathway diversity. This study demonstrates that during antigen presentation by antigen-presenting cells (APCs), *Brucella melitensis* peptides presented by MHC-II originate from a broad repertoire of proteins, not limited to surface antigens. This complex immunopeptidome, shaped by active selection mechanisms, provides diverse targets for host immune recognition. These findings establish a foundation for further investigation into the transfer of comprehensive immune information between immune cells and the elicitation of immune responses. This work also paves the way for identifying specific T-cell receptors involved in recognition and immune activation, thereby facilitating the analysis of adaptive immunity’s molecular basis. Furthermore, this study provides an innovative approach for immunopeptidome analysis, providing a crucial theoretical foundation for developing novel *Brucella melitensis* subunit vaccines.

## 1. Introduction

Building upon numerous studies on neoantigen research, Vivek P Chavda et al. [1] proposed the concept of classical immunopeptidomics. The immunopeptidome plays a critical role in the signaling processes of the adaptive immune system. Variations exist among the enzymes involved in antigen processing. Additionally, diversity is observed in the signaling proteins required for intracellular transport and their associated proteins. These components assemble with MHC molecules, forming complexes delivered to the surface of APCs for T-cell recognition. Given that CD4^+^ T-cell epitopes determine the specificity of the T-cell immune response, screening and characterizing immunopeptides constitute an essential step in developing antigenic peptide-based vaccines and immunotherapies [2]. In 2016, Mommen et al. [3] reported the identification of nearly 14,000 MHC-bound peptides from a human bone marrow monocytic leukemia cell line, capable of binding to three human leukocyte antigen (HLA) HLA-DR isoforms; this represents the largest repertoire of antigenic peptides identified to date. Additional reports on isolating and identifying MHC I/II-bound antigenic peptides have been published for human, ovine, porcine, and murine subjects, respectively [4].

*Brucella melitensis* is an intracellular parasitic bacterium capable of infecting both humans and animals. To establish an intracellular niche, it employs a four-step process: adhesion, internalization, intracellular replication, and dissemination. Within the host cell, *Brucella melitensis* undergoes complex intracellular transport, processing, and modification to generate antigenic peptides [5].

Brucellosis, the disease caused by *Brucella melitensis*, is a globally significant zoonosis. It imposes substantial economic losses and poses serious public health challenges, particularly in regions dependent on animal husbandry. Consequently, effective prevention and control of brucellosis are important for public health and the sustainable development of the livestock industry [6]. Therefore, investigating the *Brucella melitensis* immunopeptidome is vital for elucidating its pathogenesis and for brucellosis control. Furthermore, this research provides a foundation for developing more specific and efficacious recombinant subunit vaccines against brucellosis.

## 2. Results and Discussion

### 2.1. Morphological Observation of BMDCs in Mice

**Figure 1:**
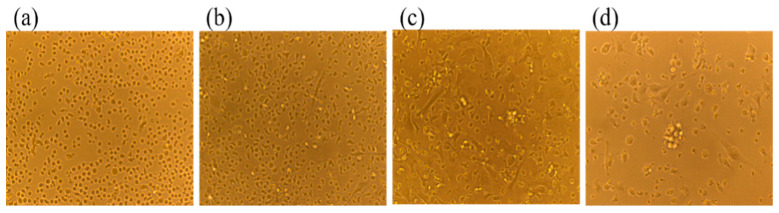
Inverted microscope observation of BMDCs morphology in mice at different times. Note: (**a**) Mouse BMDCs on day 1 of culture (×400); (**b**) Mouse BMDCs on day 3 of culture (×400); (**c**) Mouse BMDCs on day 5 of culture (×400); (**d**) Mouse BMDCs on day 7 of culture (×400).

As depicted in Figure 1, longitudinal monitoring of murine BMDCs cultures revealed progressive morphological differentiation. On day 1, homogeneous populations of small, round cells exhibited suspension growth. By day 3, most cells adhered to substrates, developing irregular morphologies with nascent pseudopodia. Day 5 cultures demonstrated significant suspension colony formation alongside increased proportions of cells exhibiting elongated, branched projections. By day 7, mature BMDCs displayed massive cellular aggregates, cytoplasm expansion, and extensive dendritic arborization. This temporal progression confirms hematopoietic precursor differentiation into functionally competent dendritic cells exhibiting characteristic morphology and antigen-presenting capacity.

### 2.2. Scanning Electron Microscope Observation of Cell Surface Morphology Before and After Brucella melitensis M5^+gfp^ Infestation

**Figure 2:**
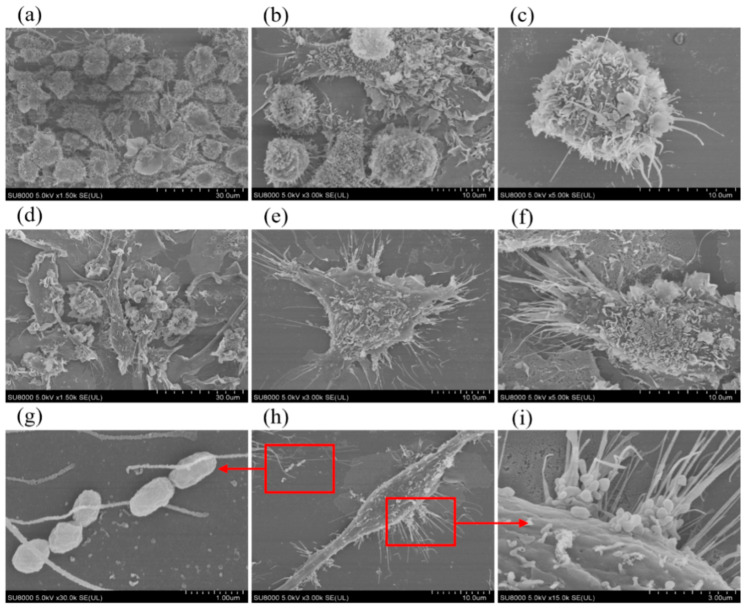
Morphological changes in cell surface before and after *Brucella melitensis M5^+gfp^* infection were observed by SEM. Note: (**a**) Mouse BMDCs on day 7 of culture (×1500); (**b**) Mouse BMDCs on day 7 of culture (×3000); (**c**) Mouse BMDCs on day 7 of culture (×5000); (**d**) Mouse BMDCs after *Brucella melitensis M5^+gfp^* infestation (×1500); (**e**) Mouse BMDCs after *Brucella melitensis M5^+gfp^* infestation (×3000); (**f**) Mouse BMDCs after *Brucella melitensis M5^+gfp^* infestation (×5000); (**g**) *Brucella melitensis M5^+gfp^* morphology (×30,000); (**h**) Mouse BMDCs after *Brucella melitensis M5^+gfp^* infestation (×3000); (**i**) *Brucella melitensis M5^+gfp^* attached to the cell surface (×15,000).

As demonstrated in Figure 2, day-7 murine BMDCs exhibited characteristic immature dendritic cell morphology: voluminous spherical cell bodies with dense surface folding and minimal dendritic branching. Following infection with *Brucella melitensis M5^+gfp^* at MOI 100:1, significant maturation was observed: cellular volume increased substantially (*p* < 0.01), surface folds decreased by approximately 40%, while dendritic processes underwent robust elongation (>200% length increase) and thickening (∼50% diameter expansion). This morphological shift toward intricate dendritic arborization indicates effective maturation of BMDCs in response to *Brucella melitensis* infection.

### 2.3. Laser Confocal Observation of Intracellular Fluorescence After Infestation

**Figure 3:**
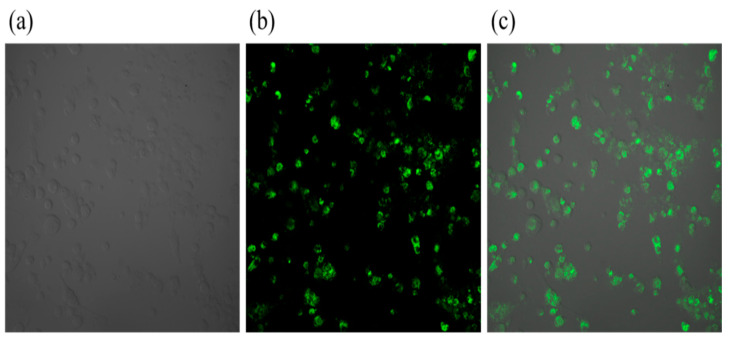
Laser confocal microscopy after *Brucella melitensis M5^+gfp^* infestation of BMDCs in mice. Note: (**a**) Represents a shaded outline of mouse BMDCs under natural light; (**b**) Represents *Brucella melitensis* containing the GFP gene, with the GFP gene protein expressed as green fluorescence; (**c**) Represents an overlay of (**a**,**b**).

As presented in Figure 3, laser scanning confocal microscopy (LSCM) analysis of *Brucella melitensis M5^+gfp^*-infected murine BMDCs revealed robust cellular infection. Superimposition of transmitted light cellular outlines with fluorescence channels demonstrated GFP-positive bacteria in >90% of cells within the field of view. High-intensity perinuclear fluorescence signal clusters (peak intensity ≥ 2.5 × 10^4^ AU) confirmed successful intracellular colonization, with bacterial load quantification indicating 185 ± 24 CFU/cell (*n* = 3 replicates). This multimodal visualization provides definitive evidence of BMDCs infection by the recombinant strain.

### 2.4. Identification of Peptide Sequences of the Brucella melitensis M5^+gfp^ Immunopeptidome

MHC-II-bound immunopeptides were isolated from hypotonically lysed cell membrane fragments and characterized by liquid chromatography-tandem mass spectrometry (LC-MS/MS). Proteomic analysis identified 289 high-confidence peptide sequences binding to MHC-II complexes, mapping to 183 unique *Brucella melitensis M5^+gfp^* source proteins (see Supplementary Table S1).

### 2.5. GO and KEGG Analysis of Proteins Corresponding to Peptide Sequences of Brucella melitensis M5^+gfp^ Immunopeptide Group

**Figure 4:**
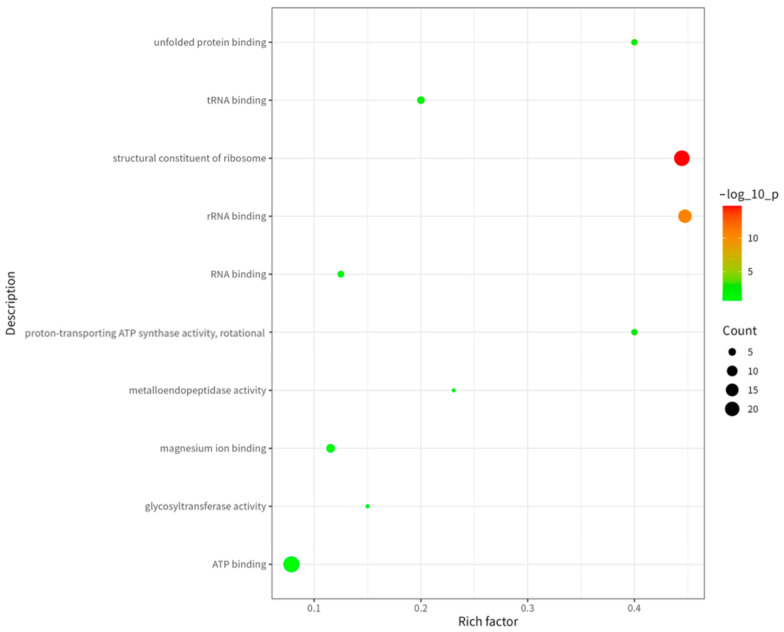
GO (MF) analysis of proteins corresponding to MHC II binding peptides in cell membrane fragment suspensions treated by hypotonic swelling.

To deeply analyze the functional characteristics of these proteins, we performed Gene Ontology (GO) analysis. As shown in Figure 4, among molecular functions (MF), the categories “ATP-binding”, “rRNA-binding” and “magnesium ion-binding” were most significantly enriched. This indicates enrichment of proteins associated with energy metabolism, protein synthesis, and enzymatic activity in the immunopeptidome. ATP-binding proteins play key roles in cellular energy metabolism [7], rRNA-binding proteins serve as essential ribosomal components for protein synthesis [8], and magnesium ion-binding proteins participate in diverse enzymatic reactions [9].

**Figure 5:**
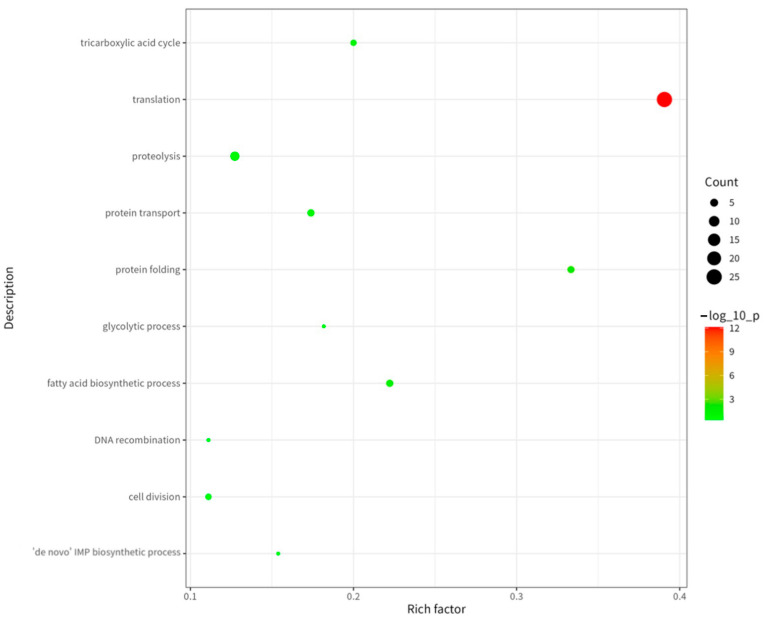
GO (BP) analysis of proteins corresponding to MHC II binding peptides in cell membrane fragment suspensions treated by hypotonic swelling.

As shown in Figure 5, among the biological processes (BPs), “translation,” “proteolysis,” and “protein folding” were the most significantly enriched processes. Translation represents the core mechanism of protein synthesis, while proteolysis facilitates protein degradation and turnover, and protein folding ensures correct conformational maturation and functionality of proteins [10]. This enrichment profile indicates that proteins comprising this immunopeptidome extensively participate in protein synthesis, degradation, and quality control pathways. These pathways are essential for maintaining intracellular protein homeostasis.

**Figure 6:**
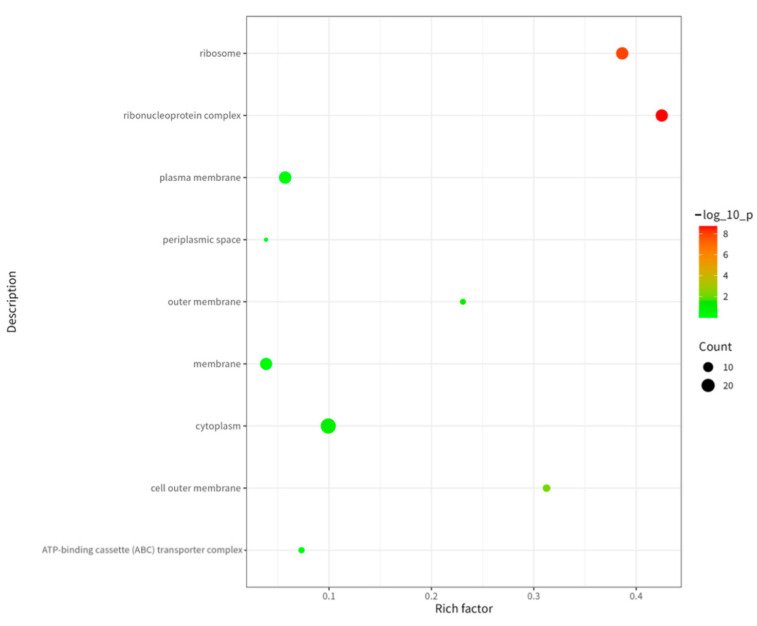
GO (CC) analysis of proteins corresponding to MHC II binding peptides in cell membrane fragment suspensions treated by hypotonic swelling.

As shown in Figure 6, cellular component (CC) analysis revealed predominant localization of immunopeptidome source proteins in the cytoplasm, ribosomes, and plasma membrane. The cytoplasm serves as the primary site for intracellular biochemical reactions, ribosomes function as the essential machinery for protein synthesis, and the plasma membrane acts as the critical interface for substance exchange and signal transduction between the cell and its environment [11]. This compartmental enrichment demonstrates broad subcellular distribution of these antigens across key functional domains.

**Figure 7:**
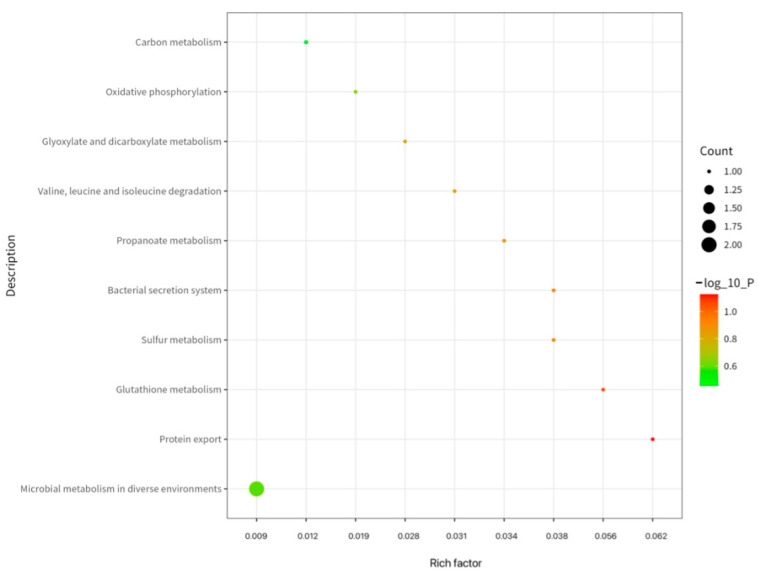
KEGG analysis of proteins corresponding to MHC II binding peptides in cell membrane fragment suspensions treated by hypotonic swelling.

As shown in Figure 7, KEGG enrichment analysis revealed the immunopeptidome’s involvement in two principal functional modules: metabolic regulation and material transport. Metabolic pathways predominated the enrichment profile (8 entries), forming the energetic and biosynthetic foundation for immune responses. Carbon metabolism and oxidative phosphorylation emerged as significantly enriched pathways, reflecting their role as primary ATP sources that support heightened immune cell activation states requiring substantial energy for proliferation and effector molecule synthesis [12]. Concurrently, glutathione metabolism establishes crucial antioxidant defense through reduced glutathione synthesis, protecting immune cells from ROS damage during inflammatory responses. Branched-chain amino acid degradation pathways provide essential amino acids while potentially modulating mTOR signaling to regulate T-cell activation [13]. whereas microbial-associated metabolic pathways suggest gut microbiota regulation of immune function through metabolite-mediated crosstalk [14].

Pathways governing transmembrane transport (2 entries) directly facilitate immune recognition and response. The antigen processing pathway for MHC class I molecules transports endogenous peptides to the endoplasmic reticulum for loading onto MHC-I complexes, enabling surface presentation to CD8^+^ T-cell [15]. Though mechanistically distinct from MHC-II presentation, these pathways collectively establish adaptive immunity’s antigen recognition network. Concurrently, bacterial secretion systems (particularly Type III) enable Gram-negative pathogens to inject effector proteins into host cells, activating NLRP3 inflammasomes and NF-κB signaling to initiate innate immune defenses [16]. This comprehensive pathway analysis, along with the GO classification detailed in Section 2.5 underscores the functional breadth of the identified immunopeptidome source proteins.

#### Functional, Structural, and Metabolic Classification of MHC-II-Bound *Brucella melitensis* Antigens

The 183 source proteins demonstrated significant functional, structural, and pathway diversity, as follows:

1. Functional classification revealed four major categories among the 183 source proteins.

(1) Redox Homeostasis (12 proteins), exemplified by superoxide dismutase (SOD) and peroxiredoxin. SOD constitutes the primary intracellular antioxidant defense through metal-dependent (Cu/Zn, Mn, or Fe) catalytic disproportionation of O_2_^−^ to O_2_ and H_2_O_2_, mitigating oxidative damage via cyclic redox reactions [17]. Peroxiredoxin decomposes H_2_O_2_ using thioredoxin reducing equivalents while maintaining redox homeostasis alongside catalase (CAT), with activity regulated through redox-state cycling [18]. (2) Energy Metabolism (28 proteins), featuring F_0_F_1_ ATP synthase subunits (α/β/γ) and glucose-6-phosphate dehydrogenase. The F_0_F_1_ complex drives ATP synthesis via proton gradient-coupled rotational catalysis [19], while glucose-6-phosphate dehydrogenase regulates the pentose phosphate pathway’s rate-limiting step, generating NADPH for biosynthetic reductions under NADP^+^/NADPH ratio control [20]. (3) Translation Machinery (35 proteins), including aminoacyl-tRNA synthetases (Tyr/Ala/Leu), ribosomal proteins (L21/S3/L7/L12), and chaperones (DnaK/GroES). Synthetases ensure translational fidelity through proofreading domains that hydrolyze mischarged tRNAs [21], ribosomal proteins (e.g., L11-stabilized 23S rRNA) maintain structural integrity [22], while the DnaK-GroES system collaborates with DnaJ/GrpE co-chaperones to prevent misfolding [23]. (4) Transport and Signaling (22 proteins), represented by TonB-dependent receptors, ABC transporters (BCAA/sugars), and FlhB. TonB systems harness proton motive force via ExbB/ExbD complexes to internalize ferric ions/vitamin B_12_ [24]. ABC transporters facilitate ATP-dependent transmembrane flux [25], and FlhB coordinates flagellar assembly for host colonization [26].

2. Metabolic pathway classification identified three core functional groups.

Carbohydrate Metabolism (18 enzymes), exemplified by pyruvate kinase which catalyzes glycolysis’ terminal ATP-generating step. This rate-limiting enzyme balances glycolytic/gluconeogenic flux through allosteric regulation by fructose-2,6-bisphosphate (F2, 6BP) [27]. (2) Amino Acid Metabolism (15 enzymes), featuring acetyl-CoA carboxylase and D-amino acid dehydrogenase. The biotin-dependent acetyl-CoA carboxylase initiates fatty acid synthesis—citrate-activated and palmitoyl-CoA-inhibited—with its biotin carboxyl carrier protein (BCCP) subunit achieving >95% carboxyl transfer efficiency via a ping-pong mechanism [28]. D-amino acid dehydrogenase synthesizes peptidoglycan precursors essential for cell wall integrity [29]. (3) Energy and Cofactor Metabolism (10 enzymes), represented by NADH: quinone oxidoreductase (Complex I) which drives proton translocation in oxidative phosphorylation [30]. And ferrochelatase (corrected terminology) that catalyzes Fe^2+^ insertion into protoporphyrin IX during heme biosynthesis, supporting cytochrome function [31].

3. Structural classification revealed three principal categories.

Outer Membrane and Secretory Proteins (25 species), exemplified by Omp31/Omp2a β-barrel channels (2.2 nm pore diameter) enabling transmembrane diffusion of small/nonpolar molecules, with aromatic residue-lined conduits facilitating passive gas transport (e.g., NO) [32]. And BamA which catalyzes β-barrel protein insertion essential for secretion systems [33]. (2) Ribosomal and Cytoplasmic Proteins (40 species), including ribosomal subunits L11 (50S) and S3 (30S) that stabilize 23S and 16S rRNA secondary structures, respectively—where L11 maintains large subunit integrity and S3 enables initiator tRNA binding critical for translational fidelity [34]. (3) Transmembrane Enzyme Complexes (18 species), featuring: Pyruvate dehydrogenase complex (E1: decarboxylase; E2: dihydrolipoyl acetyltransferase; E3: dihydrolipoyl dehydrogenase) catalyzing oxidative decarboxylation of pyruvate to acetyl-CoA via E2’s lipoyl domain achieving >95% acetyl transfer efficiency through rotational mechanics [35]. F_0_F_1_ ATP synthase whose F_0_ subunit forms a proton-conducting transmembrane channel driving ATP synthesis [36].

## 3. Materials and Methods

### 3.1. Experimental Materials and Instruments

Experimental Animals: Two hundred specific pathogen-free (SPF)-grade Kunming (KM) mice (female, 6–8 weeks old, body weight 18–22 g) were obtained from Henan Skibbes Bio-technology Co. (Zhengzhou, Henan, China). All mice were housed in individually ventilated cages (IVCs) under controlled conditions: temperature 22 ± 1 °C, humidity 45–65%, 12/12 h light/dark cycle. Autoclaved standard rodent chow and filtered water were provided ad libitum. Environmental enrichment included paper nesting material and PVC tunnels. Cage bedding was changed twice weekly by trained staff following aseptic protocols. Animals were acclimatized for 7 days prior to experiments. Ethical approval for animal use was obtained from [Approval Letter of Biology Ethics Committee of Shihezi University. A2024-424] in compliance with NIH Guide for Care and Use of Laboratory Animals (8th ed.). Pain management: No anesthetic or analgesic agents were administered during cervical dislocation euthanasia, as this method induces immediate unconsciousness per American Veterinary Medical Association (AVMA) 2023 Guidelines [37]. All procedures were performed by certified personnel within 3 min post-sacrifice to minimize tissue degradation. Bacterial Strain: The *Brucella melitensis M5^+gfp^* strain was generated by our research group through recombinant techniques and stored at −80 °C [38]. Reagents: RPMI 1640 culture medium and fetal bovine serum (FBS) were purchased from Gibco (Thermo Fisher Scientific, Waltham, MA, USA). Additionally, recombinant mouse granulocyte-macrophage colony-stimulating factor (GM-CSF) and recombinant mouse interleukin-4 (IL-4) were also sourced from Gibco (Thermo Fisher Scientific, Waltham, MA, USA). Equipment: Inverted fluorescence microscope (model XD30A, Ningbo Sunyu, Ningbo Sunny Instruments Co., Ltd., Ningbo, Zhejiang, China) Transmission electron microscope (TEM) (model H-7650, Hitachi High-Technologies Corporation, Tokyo, Japan) Scanning electron microscope (SEM) (model SU8000, Hitachi High-Technologies Corporation, Tokyo, Japan).

### 3.2. Main Reagent Preparation

#### 3.2.1. Preparation of Complete RPMI 1640 Culture Medium

A sterile, endotoxin-free 50 mL centrifuge tube was prepared. RPMI 1640 medium, fetal bovine serum (FBS; 10% *v*/*v* final concentration), and penicillin-streptomycin solution (1% *v*/*v* final concentration; 100 U/mL penicillin and 100 μg/mL streptomycin) were added to the tube and thoroughly mixed. The mixture was then filtered through a 0.22 μm sterile filter into another sterile, endotoxin-free 50 mL centrifuge tube. Subsequently, recombinant mouse interleukin-4 (IL-4) and granulocyte-macrophage colony-stimulating factor (GM-CSF) were added to achieve final concentrations of 10 ng/mL and 20 ng/mL, respectively. The medium was used fresh or stored at 4 °C.

#### 3.2.2. Preparation of 10% RPMI 1640 Culture Medium

Fetal bovine serum (FBS) and RPMI 1640 medium were combined in a 1:9 (*v*/*v*) ratio. The mixture was then filtered through a 0.22 μm sterile filter into a sterile, endotoxin-free centrifuge tube. This medium was used fresh or stored at 4 °C.

### 3.3. Experimental Replication Design

All experiments described in Section 3.4, Section 3.5, Section 3.6, Section 3.7, Section 3.8, Section 3.9 and Section 3.10 were performed with three independent biological replicates. Each replicate involved the isolation, culture, infection, and subsequent analysis of BMDCs derived from distinct cohorts of mice, processed on separate days. Data presented in the Results section represent the combined findings from these three replicate experiments. Statistical analyses (where applicable) were performed using data from all replicates.

### 3.4. Induction Culture of Mouse BMDC

Two hundred SPF-grade Kunming mice were euthanized by cervical dislocation. Femurs and tibiae were aseptically excised and flushed with PBS to isolate bone marrow cells. The resulting bone marrow cell suspension was collected and red blood cells were lysed by incubation with red blood cell lysis buffer. Following washing with PBS, the cells were resuspended in complete RPMI 1640 medium (prepared as described in Section 3.2.1) and plated into cell culture flasks with vented caps. Cells were cultured at 37 °C in a humidified 5% CO_2_ incubator. On day 3 of culture, half of the medium was replaced with fresh complete RPMI 1640 medium. On day 6, one-third of the medium was replaced with fresh complete RPMI 1640 medium. Recombinant mouse GM-CSF and IL-4 were added to the cultures on days 2 and 5 to maintain final concentrations of 20 ng/mL and 10 ng/mL, respectively. Finally, on day 7, the mature BMDCs were harvested for subsequent experiments.

### 3.5. Morphological Observation of BMDC in Mice

Cellular morphology, including adhesion status, cytoplasmic extensions, cell density, and colony formation, was assessed using an inverted fluorescence microscope on culture days 1, 3, 5, and 7.

### 3.6. Brucella melitensis M5^+gfp^ Infested Mouse BMDCs

On day 7 of culture, cells were harvested and infected with the *Brucella melitensis M5^+gfp^* strain at a multiplicity of infection (MOI) of 100:1 (bacteria–cells). Following a 1 h infection period, gentamicin was added to the culture medium to a final concentration of 50 µg/mL to kill extracellular bacteria. The infected cells were then incubated for 24 h at 37 °C. Subsequently, the cells were collected for further analysis.

### 3.7. Scanning Electron Microscope Observation of Cell Surface Morphology Before and After Infestation

BMDCs were seeded onto coverslips and divided into two experimental groups: (1) an uninfected control group, and (2) a *Brucella melitensis*-infected test group exposed to the M5*^+gfp^* strain at a multiplicity of infection (MOI) of 100:1 with 24 h incubation at 37 °C. All samples were subsequently processed for scanning electron microscopy (SEM) to evaluate bacterial adherence dynamics and infection-induced ultrastructural modifications in host cells.

### 3.8. Laser Confocal Observation of Intracellular Fluorescence After Infestation

For ultrastructural and spatial analysis, BMDCs were seeded onto glass coverslips and divided into two experimental cohorts: (1) an uninfected control group and (2) a test group infected with *Brucella melitensis M5^+gfp^* at a multiplicity of infection (MOI) of 100:1, followed by 24 h incubation at 37 °C. Subsequently, all samples underwent dual-modal imaging: scanning electron microscopy (SEM) to characterize bacterial adherence and host cell surface alterations, and LSCM to visualize intracellular bacterial localization and host cell spatial reorganization.

### 3.9. Acquisition of MHC II-Brucella melitensis M5^+gfp^ Immunopeptidome Complexes

Protein isolation via hypotonic lysis was performed as follows: Post-infection with *Brucella melitensis M5^+gfp^*, cells were harvested, washed with PBS, and resuspended in hypotonic lysis buffer (1 mM NaHCO_3_, 0.2 mM EDTA, protease inhibitor cocktail) at a 1:20 buffer-to-cell ratio. The suspension was vortexed for 1 min and incubated overnight at 4 °C. The lysate was then centrifuged at 3000× *g* for 10 min. The supernatant was carefully aspirated, yielding a pellet enriched in cell membrane fragments for subsequent analysis.

### 3.10. Isolation, Purification and Characterization of the Brucella melitensis M5^+gfp^ Immunopeptidome

Immunopeptidome samples were enriched via co-immunoprecipitation (Co-IP) using MHC-II antibodies. The captured complexes were separated and purified by capillary high-performance liquid chromatography (HPLC). Peptide mass spectrometry and bioinformatic analyses were performed by Shanghai Parsonage Biotechnology Co. (Shanghai, China). Raw mass spectrometry files were processed using MaxQuant (v1.6.1.1) with a *Brucella melitensis M5^+gfp^*-specific protein sequence database to generate theoretical spectral libraries, enabling protein identification and relative quantification. Functional annotation was conducted using R (v4.2.1) for Gene Ontology (GO) term classification, KEGG pathway enrichment, and structural domain analysis.

## 4. Conclusions

This study successfully identified 289 MHC-II-bound immunopeptides derived from 183 functionally diverse *Brucella melitensis M5^+gfp^* proteins through infection of immature BMDCs coupled with Co-IP and LC-MS/MS analysis. Systematic characterization revealed three defining features of this immunopeptidome: (1) Comprehensive functional coverage spanning antioxidant defense, energy metabolism, and protein synthesis machinery—including outer membrane proteins (e.g., BamA), secretory system components, immune signaling mediators, and intracellular folding/translocation apparatus. (2) Metabolic network interconnectivity, demonstrating tight cross-regulation between carbohydrate, amino acid, and energy cofactor pathways. (3) Structural–spatial heterogeneity with antigens distributed across outer membranes, cytoplasmic compartments, and transmembrane regions. These findings establish that APCs deploy a selective peptide screening mechanism, presenting not merely surface-derived epitopes but a multidimensional repertoire reflecting the pathogen’s physiological state. This strategy provides host immunity with layered recognition targets, activating enhanced adaptive responses. Our work provides novel insights into pathogen comprehensive antigenic profiling while revealing the immunopeptidome’s dynamic nature—providing a framework to investigate T-cell receptor interactions and serving as a rational foundation for next-generation *Brucella melitensis* subunit vaccines targeting this proteomic landscape.

## Data Availability

The datasets presented in this article are new. The data are part of an ongoing experiment. Requests can be made to access the datasets.

## Supplementary Materials

The following supporting information can be downloaded at: https://www.mdpi.com/article/10.3390/ijms26188874/s1.

## Abbreviations

The following abbreviations are used in this manuscript:

BMDCs	Bone marrow-derived dendritic cell
MHC II	Major Histocompatibility Complex Class II
Co-IP	Co-immunoprecipitation
LC-MS/MS	Liquid Chromatography-Tandem Mass Spectrometry
APC	Antigen-Presenting Cell
HLA	Human Leukocyte Antigen
MOI	Multiplicity of Infection
LSCM	Laser Scanning Confocal Microscopy
GM-CSF	Granulocyte-Macrophage Colony-Stimulating Factor
IL-4	Interleukin-4
TEM	Transmission Electron Microscope
SEM	Scanning Electron Microscope
