# Supplementary material for: Immunopeptidome Landscape During Brucella melitensis Infection in Mice
Source: Int J Mol Sci. 2025 Sep 12;26(18):8874. doi: 10.3390/ijms26188874 (PMC12469894; doi:10.3390/ijms26188874)
Supplement: Supplementary file 1 [file ijms-26-08874-s001.zip › ijms-3798502-Supplementary Materials Table S1.pdf]

Supplementary Table S1

Table 1 Summary table of MHC II binding peptide sequences and protein identification in cell membrane debris suspensions treated by hypotonic swelling

Number	Protein names	Accession	Sequence	Score	intensity
1	IMP dehydrogenase	WP_087932817.1	AAAATSVGADGFER	79.693	50395
2	urease accessory protein UreE	WP_087909543.1	AASEELYEIR	64.485	24917
3	50S ribosomal protein L21	WP_002967949.1	ADAAEGEAAKPK	102.06	34653
4	DUF2333 family protein	WP_087932945.1	ADDRFWFAYGQLYAYYGLLR	8.1875	6019
5	urease subunit alpha	WP_004682812.1	ADGELLTCEPVDVVPMAQR	81.565	93831
6	ribosomal protein S3	WP_087909567.1	ADIDYGTAEAK	91.584	286730
7	hypothetical protein	WP_002967723.1	AEANINDIQQALEK	176.18	1960300*
8	Cytochrome b N-terminal domain-containing protein	WP_004684075.1	AEIAPVEISAETKG	80.014	122160
9	Re/Si-specific NAD(P)(+)transhydrogenase subunit alpha,partial	WP_244563609.1	AETAPAAVTEK	99.815	54193
10	F0F1 ATP synthase subunit delta	WP_004684267.1	AETSSLISGVAQR	93.058	71346
11	CobS cobaltochelatase subunit CobS	WP_087932849.1	AFGVELPESAANIVLA	60.518	143080
12	YdcF family protein	WP_017750400.1	AFMQTVPAPR	71.501	31350

Number	Protein names	Accession	Sequence	Score	intensity
13	CLPB ATP-dependent chaperone ClpB	WP_124737044.1	AGELAYG TIPQLEK	100.09	278280
14	Mrp/NBP35 family ATP-binding protein,partial	WP_206118526.1	AGILDADI YGPSMPR	63.184	517080
15	glucose-6-phosphate dehydrogenase	WP_004682109.1	AGQLSDP TR	136.8	112970
16	branched-chain amino acid ABC transporter substrate-binding protein	WP_023080355.1	AGSTDDS AAVAK	82.749	37189
17	trigger factor	WP_005973381.1	AGVEVTE EELQR	170.95	271560
18	CBS domain-containing protein	WP_004683933.1	ALAAQES QAMSK	78.655	30803
19	YebC/PmpR family DNA-binding transcriptional regulator	WP_002964805.1	ALEAALG EAESIK	143.5	239950
20	glycerol-3-phosphate dehydrogenase	WP_004684843.1	ALIGTTDI AYEGR	56.359	58109
21	tonB-system energizer ExbB	WP_006136925.1	ALLADAS AGVER	87.298	43701
22	pyruvate, phosphate dikinase	WP_006152532.1	ANAETPA DAR	106.42	28922
23	DUF883 family protein	WP_014489482.1	AQDVEAQ LTATVR	145.46	136110

Number	Protein names	Accession	Sequence	Score	intensity
24	50S ribosomal protein L9	WP_004683101.1	AQLEAQNLER	154.56	415910
25	SPFH/Band 7/PHB domain protein	WP_004681235.1	AQVLEAEGNR	114.89	326820
26	hypothetical protein	WP_006140655.1	ASGAPGIGEGTSTAASTAGPGNPPSAQEQPVAVGQQALLYEER	20.016	241810
27	DsbA family protein	WP_087909557.1	ATEESAIADAVK	87.298	107680
28	NusG transcription termination/antitermination protein NusG	WP_087932883.1	ATPVDLEYGQVDKL	94.767	218820
29	FabI enoyl-ACP reductase FabI	WP_208936940.1	AVDAPDISVVKD	63.32	86028
30	NhaA Na+/H+ antiporter NhaA	WP_103053145.1	AVETEIPVVR	105.57	125570
31	30S ribosomal protein S5	WP_002964345.1	AVFETLGVQDVVAK	49.466	376920
32	tyrosine--tRNA ligase	WP_087913329.1	DAAEEAAETAR	79.974	27567
33	DUF1476 domain-containing protein	WP_004683653.1	DAEAEAYAR	154.31	92139

Number	Protein names	Accession	Sequence	Score	intensity
34	ACP S-malonyltransferase	WP_069129796.1	DVSGAAVGSAEEIEAALAALNA	44.511	291870
35	biosynthesis protein HemY	WP_004686704.1	DVVEAEVIVPAAEPVSADEPVK	108.71	41898
36	bifunctional glutamate N-acetyltransferase/amino-acid acetyltransferase ArgJ	WP_004684467.1	EATQATAEAAAK	105.2	24799
37	F0F1 ATP synthase subunit gamma	WP_087920822.1	ELIEIISGAEAL	89.298	219920
38	nucleoside-diphosphate kinase	WP_004683449.1	EVMGATNPANADEGTIR	74.326	39989
39	glycosyltransferase	WP_126981401.1	EVPPEDVNNANYFQGR	134.75	227700
40	PLP-dependent aminotransferase family protein	WP_087932979.1	FAYLSADFSNPTGETVDR	102.66	68258
41	RseP RIP metalloprotease RseP	WP_006141584.1	FIGDESETSSPVGVNESALSEEDR	2.4404	2295.1
42	acyl-CoA dehydrogenase C-terminal domain-containing protein	WP_006265927.1	FILDENGNPGTR	72.643	25159
43	pyrimidine 5'-nucleotidase	WP_087913376.1	FLGAFGVDATRAVMFENLARNLVVPK	2.1945	11248

Number	Protein names	Accession	Sequence	Score	intensity
44	methionine adenosyltransferase	WP_011005450.1	FVIGGPDG DAGLTGR	84.718	76615
45	Rho transcription termination factor Rho	WP_002965128.1	GEVISSTF DEPAAR	92.611	45908
46	sugar ABC transporter ATP-binding protein	WP_087913374.1	GIIDDIR	137.5	2405.1
47	malate dehydrogenase	WP_087909583.1	GLDIAESS PVDGFDA K	136.53	290520
48	F0F1 ATP synthase subunit B	WP_004683012.1	IAQDLEQA AR	118.5	74482
49	dihydrolipoamide acetyltransferase family protein	WP_101457630.1	IAVGSELV R	162.5	112400

50	50S ribosomal protein L14	WP_004683923.1	IISLAPEVL	128.86	449940
51	phosphoribosylaminoimidazolesuccinocarboxamide synthase	WP_002963974.1	IVVADEIS PDSAR	134.59	101330
52	alanine--tRNAligase	WP_103053188.1	KGEGVFV HIGEVTEG TAK	3.4405	101180
53	saccharopine dehydrogenase family protein	WP_005973863.1	LAADDYL DEVK	79.974	60513

Number	Protein names	Accession	Sequence	Score	intensity
54	hypothetical protein	WP_004684078.1	LADYALA TTR	131.11	63911
55	protein translocase subunit SecDF	WP_247644351.1	LAELNIDS AR	108.98	25319
56	molybdopterin-dependent oxidoreductase	WP_002966561.1	LEGPGAL AR	64.803	119700
57	acetyl-CoA carboxylase biotin carboxylase subunit	WP_014490080.1	LGIPVVPG SDGGVTD EVEAAR	68.972	42424
58	D-glycerate dehydrogenase	WP_118874249.1	LIANFGNG VDNIDVA AAARR	30.059	34344
59	GntR family transcriptional regulator	WP_101457049.1	LIDAYAV R	118.01	112170
60	thioredoxin	WP_087907738.1	LIGDPVEL ER	64.841	136940
61	PurS phosphoribosylformylglycinamidine synthase subunit PurS	WP_002963973.1	LLANTVIE DYSIAIA	103.7	163840
62	30S ribosomal protein S10	WP_002964363.1	LLDIVDPT PQTVDAL MK	50.222	915300
63	response regulator	WP_087924395.1	LLSTASGE ISR	87.298	54704

Number	Protein names	Accession	Sequence	Score	intensity
64	acyl-CoA dehydrogenase	QFP61583.1	LQAFGVTEPTSGTDTGALK	57.106	97135
65	30S ribosomal protein S7	WP_004686959.1	LSGELLDAANNR	84.17	63796
66	IclR family transcriptional regulator	WP_006153697.1	LSMVYLDVVQGEGNMTMRR	9.7521	227320
67	50S ribosomal protein L2	WP_004683925.1	LVSGACFASVGAVSNPDHGNINDGK	94.449	1630400
68	amidase	WP_103053206.1	MANLDPFNALIAK	42.348	695120
69	hypothetical protein F9L69_14770	QFP68537.1	MAQFQNSGIR	23.32	14820
70	50S ribosomal protein L4	WP_002964361.1	MDLTITTLEGK	51.927	47802
71	murein L,D-transpeptidase	WP_004686459.1	MDPLTSGMSPIR	21.464	83305
72	AGE family epimerase/isomerase	WP_103053146.1	MNDVIRKLQVEVER	33.875	67820
73	pyruvate dehydrogenase complex E1 component subunit beta	WP_101456324.1	MPIEILMPALSPTMEEGK	10.326	54289
74	nucleotidyltransferase family protein	WP_004684550.1	MRPITETMPKPLVNVAGK	52.344	1002100

Number	Protein names	Accession	Sequence	Score	intensity
75	transporter ATP-binding protein	WP_004682535.1	MSDVMTANR	9.9869	50052
76	superoxide dismutase family protein	WP_006140982.1	MYEALPTGPGK	133.13	310510
77	ubiquinol-cytochrome c reductase iron-sulfur subunit	WP_002964648.1	NANLPTDAEATDLAR	151.98	188210
78	acetyl-CoA carboxylase biotin carboxyl carrier protein	WP_002964036.1	NAVPSPMVGTAYLAPAPGAR	111.79	73346
79	DNA starvation/stationary phase protection protein Dps	WP_004686203.1	NDLPSNTK	142.19	299370
80	acyl-CoA thioesterase	WP_002964616.1	NTENEPTGMLTIR	121.29	132590
81	FlhB flagellar biosynthesis protein FlhB	WP_004681320.1	NVVRLLVANVLAIAAIAGFDLAWSRIHWR	0	5409.2
82	30S ribosomal protein S12	WP_004683928.1	PTVNQLIR	124.89	40413
83	0S ribosomal protein S2	WP_002964289.1	QASDIIADAANR	102.55	181680

Number	Protein names	Accession	Sequence	Score	intensity
84	Asp-tRNA(Asn)/Glu-tRNA(Gln) amidotransferase subunit GatB	WP_087920610.1	QIAILEDG GVIDQETR	99.671	55566
85	50S ribosomal protein L24	WP_004683922.1	QTQTQEA GIISK	149.96	272660
86	RpiB/LacA/LacB family sugar-phosphate isomerase	WP_002965776.1	SAGNVNA INEVDAK	78.692	41283
87	RidA family protein	WP_002967716.1	SAVGVAS LPLNAPVE VEAIIEVA	65.895	681250
88	50S ribosomal protein L25/general stress protein Ctc	WP_002964640.1	SEENAEG AAEEAKD GE	56.225	93024
89	50S ribosomal protein L19	WP_002964975.1	SGAGLNE NFTVR	176.6	155970
90	Hsp20 family protein	WP_002965411.1	SGDGYPP YNIER	82.831	54699
91	pyruvate kinase	WP_101419803.1	SIRCKVVS GTR	45.081	146100
92	DnaJ molecular chaperone DnaJ	WP_004684564.1	SLSVNIPA GIEDGTR	119.39	130640

Number	Protein names	Accession	Sequence	Score	intensity
93	electron transfer flavoprotein subunit beta/FixA family protein	WP_004684482.1	SPADFGA DIAPR	99.53	76537
94	Tim44/TimA family putative adaptor protein	WP_130137854.1	SSDAAPA GGGPDNV VSLPR	107.37	101170
95	30S ribosomal protein S11	WP_002964339.1	STPFAAQI AAEDCAK	82.362	180680
96	TonB-dependent receptor	WP_004685730.1	STSSVSVI DAADIER	65.574	39052
97	TIGR02300 family protein	WP_151039136.1	SYFEATVE SR	62.463	53371
98	translation elongation factor Ts	WP_002964288.1	TAAEGLV GVAASGN K	154.94	416590
99	dUTP diphosphatase	WP_002964766.1	TAASSSAP TLGIIR	127.42	88005
100	leucyl aminopeptidase	WP_011005277.1	TAEAIETT PAGVEK	103.91	83777
101	lipoprotein	WP_002964140.1	TAIPVTGT SNAAYVK	87.667	57395
102	transposase, partial	WP_206118522.1	TGDLELK QR	27.487	3764.3
103	integration host factor subunit alpha	WP_002963914.1	TGEEVPIL PR	199.02*	389890
104	integration host factor subunit beta	WP_014489310.1	TGETVDV EEK	101.65	36910

Number	Protein names	Accession	Sequence	Score	intensity
105	cytochrome c1	WP_005967752.1	TLEGLGYSPEQVK	122.57	123810
106	NADH-NADH-quinone oxidoreductase subunit J	WP_101457039.1	TPETAIEIR	114.89	60972
107	type IV secretion system protein VirB10	WP_087913357.1	TQENIPVQPGTLDGER	52.344	62543
108	2,3,4,5-tetrahydropyridine-2,6-dicarboxylate N-succinyltransferase	WP_004685944.1	TSINELLRD	72.23	74924
109	ferrochelatase	WP_005974740.1	TVPSYEAEPVYIDALAR	108.59	944840
110	leucine--tRNA ligase	WP_087907724.1	VATMLDSGAPVEIGSIEK	71.241	70291
111	hypothetical protein F9L69_10885	QFP67779.1	VFPANIKKQVASAIPILLTK	3.0979	10447
112	30S ribosomal protein S17	WP_002964353.1	VGDFVSIQESAPISK	108.35	341370
113	D-amino acid dehydrogenase	WP_101433153.1	VGGMAEVSGFTDDLPAAR	94.191	49671
114	50S ribosomal protein L1	WP_101457031.1	VGTVTTDVAAAVAASK	80.522	194490
115	S41 family peptidase	WP_118890819.1	VIAPIDDTPASK	85.29	48323

Number	Protein names	Accession	Sequence	Score	intensity
116	ABC transporter ATP-binding protein	WP_070329530.1	VIVLDEPTSALDR	96.89	76544
117	hypothetical protein	WP_004683155.1	VIYQAALTDTTR	111.06	119570
118	polyribonucleotide nucleotidyltransferase	WP_124737052.1	VLAVVENDLR	64.654	38318
119	peroxiredoxin	WP_004686034.1	VLDALQTDELCPCNR	183*	213890
120	50S ribosomal protein L20	WP_002965185.1	VLDQAAGFR	67.903	46806
121	sigma-54 dependent transcriptional regulator	WP_006144450.1	VLVDQQFER	103.56	38249
122	virB8 family protein	WP_002966517.1	VNAQTGAPDILTSLDEK	101.43	476170
123	30S ribosomal protein S13	WP_002964340.1	VNQLSDAEVLQIR	132.78	116590
124	methylated-DNA--[protein]-cysteine S-methyltransferase	WP_101415739.1	VRALLDGASPDFSDTPLALDSVPDLNRR	0.63148	9984.5
125	GumC family protein	WP_087932900.1	VVGTELTPGQLPTDASLAVAESQAR	28.951	68183

Number	Protein names	Accession	Sequence	Score	intensity
126	aldehyde dehydrogenase family protein	WP_118874233.1	YFENTSPVNGQVLCEVAR	109.22	124850
127	beta-ketoacyl-ACP synthase I	WP_011005448.1	YNDTPSTASR	107.15	24999
128	50S ribosomal protein L7/L12	WP_002964371.1	AQLEAAGAK	145.65	943350
			DLVEGAPK	119.29	118800
129	DUF1013 domain-containing protein	WP_002964823.1	AIADGEASQGIK	103.7	148250
			GLDPVITGQLSR	99.788	112870

130	hypothetical protein	WP_002964396.1	AATAQITPEEK	106.42	83176
			QAQEQTNATLK	109.44	31295
131	GrpE nucleotide exchange factor GrpE	WP_002965419.1	AEAAADEAEGEVDETANR	112.51	260890
			ALDAIPADALEADSNLK	81.92	635400
132	30S ribosomal protein S1	WP_002965274.1	IENALGEAVLSR	112.13	120460
			SEIVQNLEEGQVVEGVVK	111.91	122770
133	catalase	WP_002966234.1	DAHGYDANTIALNEK	62.357	47115
			EPPLCISGNADR	75.294	55903

Number	Protein names	Accession	Sequence	Score	intensity
			IGNDDYSQPR	107.57	65292
			TGNPDAYYEPNSFNGPVEQPSAK	18.699	72254
134	co-chaperone GroES	WP_002966386.1	EKPQEGEVVAAGAGAR	154.69	3179400*
			IGGEDLLIMK	136.27	1799900*
			TAGGIIIPDTAK	125.74	3072300*
135	Hsp20 family protein	WP_002966528.1	ADETASDVGEVLYR	112.15	100090
			AGSGTAQPIEAK	90.108	112740
136	molecular chaperone DnaK	WP_002969217.1	DAEANAEADKK	213.53*	302330
			IINEPTAAALAYGLDK	120.47	6082300*
			IQASGGLSDADIEK	145.91	901660
			SQTFSTAEDNQSAVTIR	105	336520
			TTPSIIAFTDGDER	157.97	2674400*
			VIENAEGAR	172.15	1959400*
			VIGIDLGTTNSCVAVMDGK	130.07	879510
137	invasion associated locus B family protein	WP_004682929.1	DTTACVMR	70.912	72075

Number	Protein names	Accession	Sequence	Score	intensity
			QEQSSAQAGQR	176.79*	221830
			VLTAELR	160.69	404730
138	3-deoxy-7-phosphoheptulonate synthase class II	WP_004683758.1	NVTECTGGAR	105.52	52716
			QVPFYPDAQALNDVEAR	68.809	143490
139	type I glyceraldehyde-3-phosphate dehydrogenase	WP_004684167.1	EVEVAGDTIDVGYGPIK	59.57	42089
			MSDTAVALGK	68.224	46151

140	F0F1 ATP synthase subunit beta	WP_004684261.1	AALVYGQMNEPPGAR	65.574	81811
			DTGEPIMVPVGVETLGR	198.32*	2400800*
			FTQAGSEVSALLGR	210.44*	1545200
			GIYPAVDPLDSTSR	155.11	854320
			IMNVIGEPVDEAGPIK	153.95	2348900*
			TIAMDATEGLVR	154.56	424300
			TTAAAEAKPAAK	105.2	176670
			TTAAAKPAATK	97.965	31183
141	DegQ family serine endoprotease	WP_004685513.1	DIAASLGLAEEK	172.27	112580
			GAIVASPQDDGPAAK	91.937	194050

Number	Protein names	Accession	Sequence	Score	intensity
			SGDVIVSV NNQTVK	88.596	73719
142	2-oxoglutarate dehydrogenase complex dihydrolipoyllysine-residue succinyltransferase	WP_004685222.1	EEAKPAA AAPAAAP VASASSGP AMQPAPA AAK	28.673	102470
			EGDTVEV GELLGQIS SDGAAVA AAPAQK	228.51*	1606800
			LLAESGLS ADQVEGS GK	224.24*	263980
143	Outer membrane protein Omp31	WP_004685017.1	LGYTATE R	170.06	2321700*
			NLVDVDN SFLESK	167.12	31213000*
144	50S ribosomal protein L15	WP_004685698.1	AGGSIKLP EAAAE	73.885	318440
			SGVSINGF EGGQMPI YR	85.288	57969
145	25 kDa outer-membrane immunogenic protein	WP_004685536.1	LNNGLDD ESK	138.98	202740
			LTDNILGR	175.75	67088
			NYDLAGT TVR	182.53*	241610
			QGFEGSLR	152.41	2504700*
			VGWTAGA GLEAK	84.658	196680
146	30S ribosomal protein S8	WP_004686960.1	EQNVGGE LLCR	134.66	259410

Number	Protein names	Accession	Sequence	Score	intensity
			YYEGVPVIR	117.17	224860
147	protein translocase subunit SecD	WP_005968113.1	QEGGEEAQTAAVNK	70.942	32357
			TAEEGAIAQK	101.72	41343
			TNEPIVSFR	114.89	50111
			VVGDAVVVTIPDADQR	109.6	28469

148	30S ribosomal protein S9	WP_004686488.1	TEAAAPVDVQK	109.29	493490
			VESINSLEELGTVAK	174.24	1760700*
149	outer membrane protein assembly factor BamA	WP_006137216.1	EFDLNEGDAFNQVMVQR	110.66	75641
			NDVYDEGR	121.74	64878
			STGEFSIGGGYTTGGESPGAQVEAAITER	35.923	121770
			TVDLGQGR	138.37	65234
			VNVVYEINEGSR	125.97	113420
			YTFGDVSVESTVDGVDTQALDR	72.175	554610

Number	Protein names	Accession	Sequence	Score	intensity
150	outer membrane protein Omp2a	WP_006142025.1	GGDDVYS GTDR	125.82	273530
			NTVAEDN AWGGIVR	97.813	81677
			VSTGSETE LGTLK	104.79	271150
151	peptidoglycan-associated lipoprotein Pal	WP_006267420.1	ADAQQTL SK	126.62	447550
			AVTVLNG AGR	100.45	59382
			EYNLALG QR	134.66	70672

152	DUF4167 domain-containing protein	WP_006152741.1	AEQPQPAI EAPAAAA R	56.225	40580
			VEEAAPA VVEAAPV EASAEEA GAPR	62.193	136590
153	HflK FtsH protease activity modulator HflK	WP_006213848.1	EVADAFD EVQR	91.867	68043
			VVQDAEG EAQR	147.2	56366
154	threonine--tRNA ligase	WP_006144467.1	LCEEATPP DLLR	93.111	54367
			SNTVSLQF PDGSVR	89.548	77157
155	50S ribosomal protein L3	WP_029077456.1	AGAKAEA AATEGGE	67.726	26121
			VTTQNIEV VSTDSDR	141.73	180860
156	fumarylacetoacetate hydrolase family protein	WP_029077452.1	AGDVVEL GIEGLGSQ K	93.011	158360

Number	Protein names	Accession	Sequence	Score	intensity
			ATSAIVGPNDDLVIPR	140.78	251680
157	FtsH ATP-dependent zinc metalloprotease FtsH	WP_014489490.1	EISYSQFIDDVSNGR	112.86	86853
			NVPLAPNVDLK	74.267	230760
			QVVVPNPDIVGR	75.652	68570

158	DNA-directed RNA polymerase subunit alpha	WP_065874193.1	AEDAPIGLIPVDSLYSPVR	147.7	796950
			GYVPADCNR	108.09	63104
159	elongation factor Tu	WP_040120183.1	AYDQIDAAPEER	188.04*	977540
			TVGAGIVSSIIE	82.452	702220
			VGEEVEIVGIK	150.27	1641400
160	D-ribose ABC transporter substrate-binding protein	WP_069129862.1	DNVDKYNSPFVLEQ	89.805	228680
			YVELFGAPSDNNAATR	106.49	230480
161	mitofilin family membrane protein	WP_075630298.1	AIGEWEQLPADAK	85.622	73073
			APVTGASDNTTGEAPASSGQ	57.802	47702
			ATPAAEPVGGFSGK	129.82	187940

Number	Protein names	Accession	Sequence	Score	intensity
			NPVTINLD PSEVK	93.096	55805
162	TonB-dependent receptor	WP_087910434.1	IAGLPNYD SQK	82.645	202660
			NGDGTEIL GTEPAAR	72.089	45535
			NNAFYTD PTFSPGR	124.67	115550
163	DNA-directed RNA polymerase subunit beta'	WP_087920739.1	GTPVNQG EAVGVIA AQSIGEPG TQLTMR	28.215	172070
			IIIEPNDDT IEPVEYLIP K	79.639	1188500
			SALTCETR	114.78	55419
164	peroxiredoxin	WP_087913324.1	SLNIEEQP GQAVTSA ASALLAQ L	97.203	589390
			TADGVTE MTTDDVF K	102.4	136120
			YSAIVEDG VVK	90.15	290620
167	OmpA family protein	WP_087924404.1	AASVASY LDSQGIDP R	83.397	50696
			AQLQGTG VSVTR	83.862	42503
168	tetratricopeptide repeat protein	WP_087932740.1	AIQLNPQY DAAYIGR	62.408	55976
			AISLNSTA PEPYNGR	80.763	60982

Number	Protein names	Accession	Sequence	Score	intensity
169	sodium-translocating pyrophosphatase	WP_087932724.1	ATDALDA VGNTTK	159.83	73100
			SFEDGFTD ADGVK	72.006	21279
170	phosphopyruvate hydratase	WP_087932779.1	SGETEDST IADLAVAT NCGQIK	94.728	429270
			TAIIDIVGR	130.22	966470
171	chaperonin GroEL	WP_087932869.1	AAVEEGIV AGGGTAL LR	187.78*	8291800*
			ENTTIVDG AGQK	152.11	235080
			GVNADQE AGINIVR	261.65*	1673400*
			GYLSPYFV TNPEK	81.865	897180
			INTSEEVA QVGTISAN GEAEIGK	73.389	68255
			KINTSEEV AQVGTISA NGEAEIGK	119.01	2723000*
			MIAEAMQ K	113.1	403230
			QITTNAGE EASVIVGK	136.38	662630
			TAETELEV VEGMQFD R	342.62*	5666600*

Number	Protein names	Accession	Sequence	Score	intensity
			TNDTAGDGTTTATVLGQAIVQEGAK	189.81*	1472700
			VDDALNATR	145.16	672800
			VGGATEVEVK	183.65*	1342600
			VGNEGVITVEEAK	227.37*	3522900*
172	membrane protein insertase YidC	WP_101415741.1	DAAIAQSPR	117.4	45161
			YQSDLLSAPLTVAPGQSQK	70.622	63322
173	NAD-glutamatedehydrogenase	WP_101419116.1	LVDGLPEPR	73.616	14122
			NAVIVPVGAK	70.919	66346
			SSAETDAQVGDR	132.91	84871
			VIGEGANLGVTQR	106.29	62053
174	aminopeptidase	WP_101419834.1	CFVDGATLTPEEIR	100.72	119800
			LAIAADNPMLLSTQDAGK	52.555	32388
			VLDTDEGACR	182.39*	36141

Number	Protein names	Accession	Sequence	Score	intensity
			YANAPDE SFDR	127.4	116460
175	pyruvate dehydrogenase complex dihydrolipoamide acetyltransferase	WP_101419815.1	DVEAALA SGGAK	83.862	156020
			EAAAPAA APAPAR	169.44	146850

176	flagellar biosynthetic protein FliO	WP_101433138.1	IEPVVPFV AQEVR	98.353	1803600*
			LSVMDAA AVDSR	103.22	58017
177	NADP-dependent isocitrate dehydrogenase	WP_101457606.1	CATITPDE AR	176.79	139990
			DATNDQV TIDAANAI K	89.403	120930
178	porin family protein	WP_118874251.1	DFSVVDG DLSVEAR	156.51	143610
			NSVSLTD GIESIGVS QSK	222.29*	1157200
			STLSDEDT SIR	226.47*	3444100*
179	DUF1775 domain-containing protein	WP_110506816.1	VGDLVITD PSVR	120.45	264350
			VTVDLPE GFIMAQP QAK	78.653	1270900

Number	Protein names	Accession	Sequence	Score	intensity
180	DNA-directed RNA polymerase subunit beta	WP_124737035.1	AEAPFVGTGMEPIVAR	151.55	842660
			DIPNVSEEALK	87.913	167490
			DSGAAIAAR	154.01	285950
			SLGLNVELDDTR	129.7	115300
181	ribosome recycling factor	WP_130163227.1	ASASLLEPITIEAYGSTMPINQVANISVPESR	65.893	341120
			DSVISQDESR	168.27	231410
182	25 kDa outer-membrane immunogenic protein	WP_247876854.1	DYGFDDAAVTNNFK	53.569	119050
			GEFEGTGFSQSK	114.4	239160
			NIDFLDENNAAATFENR	270.45*	1605800
183	elongation factor G, partial	WP_247872136.1	LAAEDPSFR	130.22	89222
			QSGGSGQFAR	109.11	35708

Note: *Top 20 for peptide LFQ and score.
